# Increased neutrophil percentage-to-albumin ratio is associated with all-cause mortality in patients with severe sepsis or septic shock

**DOI:** 10.1017/S0950268820000771

**Published:** 2020-04-02

**Authors:** Yuqiang Gong, Diwen Li, Bihuan Cheng, Binyu Ying, Benji Wang

**Affiliations:** Department of Anesthesiology, Critical Care and Pain Medicine, The Second Affiliated Hospital and Yuying Children's Hospital of Wenzhou Medical University, Wenzhou 325000, Zhejiang, China

**Keywords:** Intensive care unit, mortality, neutrophil percentage-to-albumin ratio, septic shock, severe sepsis

## Abstract

There has been no study exploring the prognostic values of neutrophil percentage-to-albumin ratio (NPAR). We hypothesised that NPAR is a novel marker of inflammation and is associated with all-cause mortality in patients with severe sepsis or septic shock. Patient data were extracted from the MIMIC-III V1.4 database. Only the data for the first intensive care unit (ICU) admission of each patient were used and baseline data were extracted within 24 h after ICU admission. The clinical endpoints were 30-, 90- and 365-day all-cause mortality in critically ill patients with severe sepsis or septic shock. Cox proportional hazards models and subgroup analyses were used to determine the relationship between NPAR and these clinical endpoints. A total of 2166 patients were eligible for this analysis. In multivariate analysis, after adjustments for age, ethnicity and gender, higher NPAR was associated with increased risk of 30-, 90- and 365-day all-cause mortality in critically ill patients with severe sepsis or septic shock. Furthermore, after adjusting for more confounding factors, higher NPAR remained a significant predictor of all-cause mortality (tertile 3 *vs*. tertile 1: HR, 95% CI: 1.29, 1.04–1.61; 1.41, 1.16–1.72; 1.44, 1.21–1.71). A similar trend was observed in NPAR levels stratified by quartiles. Higher NPAR was associated with increased risk of all-cause mortality in critically ill patients with severe sepsis or septic shock.

## Introduction

Sepsis is a syndrome of physiological, pathological and biochemical abnormalities induced by infection [1]. Septic shock causes circulatory and metabolic abnormalities, leading to increased mortality in hospitalised patients, especially in intensive care unit (ICU) patients [2, 3]. Studies showed that once sepsis advanced to septic shock, the mortality rate rose from 25% to 52%, despite adoption of therapeutic strategies according to international sepsis guidelines [4, 5]. Given the poor prognosis of septic shock in critical illness, researchers have found multiple risk factors predicting the prognosis of these patients, with the aim of early intervention to reduce mortality [6, 7]. Nevertheless, the mortality caused by sepsis remains high.

Neutrophils play crucial roles in the innate cellular immune system. Previous studies suggested that early higher neutrophil counts correlated with increased sepsis severity [8, 9], and neutrophil percentage was predictive of bloodstream infection [10]. Albumin is a medium-sized molecule that is the most abundant protein in human plasma. For a variety of physiological mechanisms, albumin is indispensable. It has a variety of functions, including serving as a major buffer, extracellular antioxidant, immunomodulator, antidote and transporter in plasma [11, 12]. Increased capillary leakage of albumin is one of the features of SIRS [13]. This means that lower albumin levels correlate with severe systemic inflammation and organ failure [14]. Moreover, several studies demonstrated that low albumin levels correlated with adverse clinical outcomes [11, 15].

Recently, the neutrophil-albumin ratio has been identified as a prognostic predictor in patients with rectal cancer and palliative pancreatic cancer [16, 17]. Nevertheless, to our knowledge, no previous study has focused on the neutrophil percentage-to-albumin ratio (NPAR). In this study, we hypothesised that NPAR is a novel marker of inflammation associated with all-cause mortality in patients with severe sepsis or septic shock.

## Methods

### Data source

Similar to our previous studies, we followed the methods of Wang *et al*., 2019 [18, 19]. The study was based on a publicly accessible clinical database called the Multiparameter Intelligent Monitoring in Intensive Care III version 1.4 (MIMIC-III v1.4). It includes approximately 40 000 critical care patients at the Beth Israel Deaconess Medical Center (Boston, USA) from 2001 to 2012 [20]. The demographics, vital signs, laboratory tests, medications, nursing progress notes and other clinical variables were recorded in this database. The project was approved by the institutional review boards of the Massachusetts Institute of Technology (MIT) and Beth Israel Deaconess Medical Center (BIDMC). To apply for access to the database, we passed the Protecting Human Research Participants exam and obtained a certificate (No. 6182750). Health data of all patients in this database were de-identified; therefore, informed consent was waived.

### Population selection criteria

Inclusion criteria included: (1) adult patients (⩾18 years) diagnosed with severe sepsis or septic shock; (2) hospitalisation in the ICU at first admission for more than 2 days. Exclusion criteria were as follows: (1) no neutrophil percentage and albumin measured during ICU stay and (2) more than 5% of individual data missing. Severe sepsis was defined as systemic inflammatory response syndrome caused by infection combined with acute organ dysfunction (SOFA scoring system). Septic shock was defined as the presence of infection and systemic inflammatory response syndrome as defined in severe sepsis as well as the presence of arterial hypotension with a systolic blood pressure ⩽90 mmHg or a mean arterial blood pressure ⩽70 mmHg for at least 2 h or administration of a vasopressor (dopamine ⩾5 μg/kg/min; norepinephrine, epinephrine, phenylephrine, or vasopressin in any dosage) to maintain systolic blood pressure ⩾90 mmHg or mean arterial blood pressure ⩾70 mmHg despite adequate fluid loading [21].

### Data extraction

Structured Query Language (SQL) with the PostgreSQL tool (version 9.6) was used to extract the data from MIMIC-III. Extracted data included demographics, vital signs, comorbidities, laboratory parameters and others upon admission. We extracted comorbidities, including congestive heart failure (CHF), coronary artery disease (CAD), atrial fibrillation (AFIB), stroke, renal disease, liver disease, pneumonia, malignancy, respiratory failure, chronic obstructive pulmonary disease (COPD) and acute respiratory distress syndrome (ARDS). The laboratory parameters included neutrophil percentage, albumin, bicarbonate, anion gap, creatinine, bilirubin, chloride, glucose, haematocrit, haemoglobin, platelet, sodium, potassium, blood urea nitrogen (BUN), white blood cell (WBC), lactate, prothrombin time (PT), international normalised ratio (INR) and activated partial thromboplastin time (APTT). Sequential organ failure assessment (SOFA) scores [22] and simplified acute physiology scores II (SAPSII) [23] were calculated for each patient. Age, gender, ethnicity, systolic blood pressure (SBP), diastolic blood pressure (DBP), mean blood pressure (MBP), respiratory rate, temperature, heart rate, SPO_2_, renal replacement therapy, vasopressor use and length of stay in the ICU were extracted. All baseline data were extracted within 24 h after ICU admission; 30-, 90- and 365-day all-cause mortality were the endpoints.

### Statistical analysis

Continuous variables were presented as mean ± standard deviation (SD) or medians and interquartile range, and were tested by One-Way ANOVA (normal distribution) and Kruskal–Wallis H (skewed distribution). Categorical data were summarised as number or percentage and were compared using the chi-squared test. The association between NPAR levels and 30-, 90- and 365-day all-cause mortality was evaluated using Cox proportional hazards models. The results of the multivariate analysis were presented as hazard ratios (HRs) with 95% confidence intervals (CIs).

Two multivariate models were used to evaluate the prognostic values of NPAR for each endpoint. In model I, covariates were only adjusted for age, ethnicity and gender. In model II, we further adjusted for age, gender, ethnicity, SBP, DBP, temperature, SPO_2_, anion gap, bicarbonate, chloride, haemoglobin, lactate, platelet, APTT, PT, BUN, WBC, vasopressor use, atrial fibrillation, liver disease, respiratory failure, SOFA and SAPSII. We selected these confounders based on a change in effect estimate of more than 10%. The receiver operating curve (ROC) test was performed to measure the sensitivity and specificity of NPAR and other variables (SOFA score, albumin and neutrophils percentage) and calculated the area under the curve (AUC) to ascertain the quality of NPAR as a predictor of 365-day all-cause mortality.

Subgroup analysis of the associations between NPAR and 90-day all-cause mortality was performed to examine whether the effect of the NPAR differed across various subgroups. All statistical analyses were performed using EmpowerStats version 2.17.8 (http://www.empowerstats.com/cn/, X&Y solutions, Inc., Boston, MA) and R software version 3.42; *P* < 0.05 was considered statistically significant.

## Results

### Subject characteristics

A total of 2166 patients were eligible for this analysis. The demographic characteristics of participants stratified by NPAR tertiles are summarised in Table 1. Of these patients, there were 1204 (55.6%) men and 1595 (73.6%) white. According to NPAR levels, patients were divided into three groups (tertile 1: NPAR <24.4; tertile 2: NPAR ⩾24.4, <31.4; tertile 3: NPAR ⩾31.4), and the number of patients in each group was 722. Patients in the high tertile of NPAR (NPAR ⩾31.4) were more likely to use vasopressor, to report a history of malignancy, had lower SBP, DBP, MBP, haematocrit, haemoglobin and had higher values of chloride, BUN, WBC and mortality.

**Table 1.** Characteristics of the study patients according to NPARs

Characteristics	NPARs			
	<24.4 (*n* = 722)	⩾24.4, <31.4 (*n* = 722)	⩾31.4 (*n* = 722)	*P* value
Age, years	64.9 ± 16.5	67.3 ± 16.0	66.1 ± 16.6	0.022
Gender, *n* (%)				0.001
Female	287 (39.8)	318 (44.0)	357 (49.4)	
Male	435 (60.2)	404 (56.0)	365 (50.6)	
Ethnicity, *n* (%)				0.070
White	530 (73.4)	527 (73.0)	538 (74.5)	
Black	89 (12.3)	73 (10.1)	59 (8.2)	
Other	103 (14.3)	122 (16.9)	125 (17.3)	
NPAR	18.2 ± 6.0	27.8 ± 2.0	39.1 ± 7.9	<0.001
SBP, mmHg	111.1 ± 14.6	110.7 ± 14.7	109.3 ± 14.6	0.048
DBP, mmHg	58.5 ± 9.9	57.2 ± 9.5	56.7 ± 10.2	0.002
MBP, mmHg	74.1 ± 10.2	72.9 ± 9.6	72.5 ± 10.7	0.008
Heart rate, beats/min	92.3 ± 19.2	90.9 ± 16.9	93.1 ± 16.9	0.058
Respiratory rate, beats/min	21.2 ± 4.8	21.3 ± 4.5	21.4 ± 4.7	0.753
Temperature, °C	36.9 ± 0.9	36.8 ± 0.7	36.7 ± 0.8	<0.001
SPO2, %	96.1 ± 4.6	96.7 ± 3.0	96.5 ± 4.1	0.016
Comorbidities, *n* (%)				
Congestive heart failure	117 (16.2)	183 (25.3)	116 (16.1)	<0.001
Coronary artery disease	129 (17.9)	154 (21.3)	119 (16.5)	0.051
Atrial fibrillation	192 (26.6)	248 (34.3)	230 (31.9)	0.005
Stroke	41 (5.7)	39 (5.4)	39 (5.4)	0.965
Renal disease	117 (16.2)	140 (19.4)	121 (16.8)	0.234
Liver disease	95 (13.2)	90 (12.5)	91 (12.6)	0.916
Pneumonia	321 (44.5)	304 (42.1)	271 (37.5)	0.025
Malignancy	165 (22.9)	121 (16.8)	182 (25.2)	<0.001
Respiratory failure	414 (57.3)	416 (57.6)	447 (61.9)	0.141
COPD	20 (2.8)	26 (3.6)	14 (1.9)	0.157
ARDS	19 (2.6)	21 (2.9)	20 (2.8)	0.950
Laboratory parameters				
Neutrophil percentage, %	62.4 ± 23.9	81.8 ± 9.4	85.8 ± 7.8	<0.001
Albumin, g/dl	3.4 ± 0.7	3.0 ± 0.4	2.3 ± 0.4	<0.001
Bicarbonate, mg/dl	19.1 ± 5.5	19.3 ± 5.6	19.1 ± 5.7	0.618
Anion gap, mmol/l	14.3 ± 4.2	14.2 ± 4.3	13.4 ± 4.0	<0.001
Creatinine, mEq/l	1.7 ± 1.5	1.9 ± 1.7	1.7 ± 1.5	0.006
Bilirubin, mg/dl	2.3 ± 5.4	2.2 ± 4.8	2.7 ± 5.1	0.151
Chloride, mmol/l	100.8 ± 7.5	101.5 ± 8.1	103.2 ± 8.1	<0.001
Glucose, mg/dl	144.3 ± 51.4	145.3 ± 48.4	141.9 ± 50.3	0.407
Haematocrit, %	29.5 ± 6.5	29.3 ± 5.5	27.5 ± 5.5	<0.001
Haemoglobin, g/dl	9.9 ± 2.2	9.8 ± 1.9	9.2 ± 1.8	<0.001
Platelet, 10^9^/l	154.1 ± 101.7	198.3 ± 133.7	200.5 ± 139.3	<0.001
Sodium, mmol/l	135.9 ± 5.7	135.9 ± 6.6	136.1 ± 6.6	0.770
Potassium, mmol/l	3.8 ± 0.6	3.8 ± 0.6	3.7 ± 0.7	0.021
BUN, mg/dl	32.0 ± 22.0	36.1 ± 24.8	38.3 ± 29.3	<0.001
WBC, 10^9^/l	9.8 ± 12.1	12.0 ± 7.5	14.6 ± 9.5	<0.001
Lactate, mmol/l	2.4 ± 2.0	1.9 ± 1.7	2.0 ± 1.6	<0.001
PT, second	16.1 ± 5.0	16.8 ± 6.8	16.4 ± 5.2	0.091
APTT, second	33.0 ± 10.5	33.5 ± 11.3	34.9 ± 12.4	0.005
INR	1.5 ± 0.6	1.6 ± 0.9	1.5 ± 0.7	0.097
Scoring systems				
SOFA	7.7 ± 4.2	7.2 ± 4.0	7.7 ± 4.0	0.031
SAPSII	46.4 ± 17.1	46.5 ± 15.7	48.3 ± 16.7	0.053
Renal replacement therapy, *n* (%)	98 (13.6)	110 (15.2)	113 (15.7)	0.501
Vasopressor use, *n* (%)	445 (61.6)	452 (62.6)	494 (68.4)	0.015
ICU LOS, day	6.8 ± 8.4	7.5 ± 9.2	7.9 ± 9.5	0.047
30-day mortality, *n* (%)	188 (26.0)	177 (24.5)	247 (34.2)	<0.001
90-day mortality, *n* (%)	239 (33.1)	251 (34.8)	325 (45.0)	<0.001
365-day mortality, *n* (%)	305 (42.2)	337 (46.7)	403 (55.8)	<0.001

NPAR, neutrophil percentage-to-albumin ratio; SBP, systolic blood pressure; DBP, diastolic blood pressure; MBP, mean blood pressure; COPD, chronic obstructive pulmonary disease; ARDS, acute respiratory distress syndrome; BUN, blood urea nitrogen; WBC, white blood cell; PT, prothrombin time; APTT, activated partial thromboplastin time; INR, international normalised ratio; SOFA, Sequential Organ Failure Assessment; SAPSII, Simplified Acute Physiology Score II; ICU, intensive care unit; LOS: length of stay.

The statistical methods used for comparisons were the One-Way Anova (normal distribution), Kruskal–Wallis H (skewed distribution) test and chi-square tests (categorical variables).

### NPAR as a predictor of the clinical endpoints

**Fig. 1.**
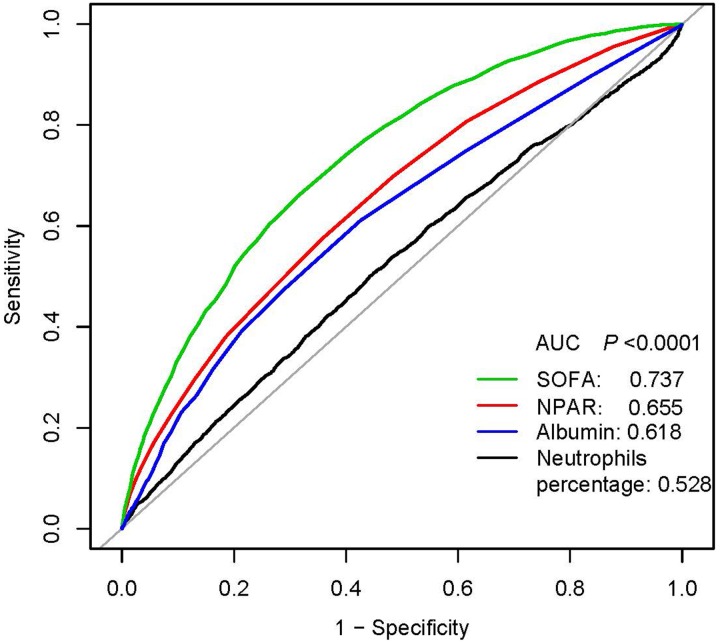
ROC curves for the prediction of 365-day all-cause mortality in critically ill patients with severe sepsis or septic shock. The AUCs for NPAR, albumin, neutrophils percentage and SOFA scores were 0.655, 0.618, 0.528 and 0.737, respectively.

In multivariate analysis, we stratified NPAR levels by tertiles and quartiles, to assess whether NPAR was associated with 30-, 90- and 365-day all-cause mortality (Table 2). In model I, after adjustments for age, ethnicity and gender, higher NPAR was associated with increased risk of all-cause mortality. In model II, after adjusting for age, gender, ethnicity, SBP, DBP, temperature, SPO_2_, anion gap, bicarbonate, chloride, haemoglobin, lactate, platelet, APTT, PT, BUN, WBC, vasopressor use, AFIB, liver disease, respiratory failure, SOFA and SAPSII, higher NPAR was still significantly associated with 30-, 90- and 365-day all-cause mortality compared with the low NPAR levels (tertile 3 *vs*. tertile 1: HR, 95% CI: 1.29, 1.04–1.61; 1.41, 1.16–1.72; 1.44, 1.21–1.71). A similar trend was observed in NPAR levels stratified by quartiles; high-NPAR levels were also independently associated with these clinical endpoints (quartile 4 *vs*. quartile 1: HR, 95% CI: 1.37, 1.07–1.77; 1.43, 1.15–1.79; 1.41, 1.16–1.73). The generated ROC curves were shown in Figure 1. The AUCs for NPAR, albumin, neutrophils percentage and SOFA scores were 0.655, 0.618, 0.528 and 0.737, respectively. The findings indicated that NPAR was a better predictor of 365-day all-cause mortality than either albumin or neutrophil percentage alone (*P* < 0.0001). Moreover, we compared NPAR with neutrophil: lymphocyte (NLR) and lymphocyte in the supplementary materials, and the AUCs for NPAR, NLR and lymphocyte were 0.655, 0.646 and 0.576, respectively.

**Table 2.** HRs (95% CIs) for all-cause mortality across groups of NPARs

NAR	Non-adjusted		Model I		Model II	
	HR (95% CIs)	*P* value	HR (95% CIs)	*P* value	HR (95% CIs)	*P* value
*30-day all-cause mortality*						
Tertiles						
<24.4	1.0 (ref)		1.0 (ref)		1.0 (ref)	
⩾24.4, <31.4	0.92 (0.75, 1.13)	0.4356	0.87 (0.70, 1.06)	0.1731	1.02 (0.82, 1.28)	0.8347
⩾34.4	1.37 (1.13, 1.66)	0.0011	1.35 (1.12, 1.64)	0.0020	1.29 (1.04, 1.61)	0.0229
*P* trend	0.0004		0.0006		0.0190	
Quartiles						
<22.5	1.0 (ref)		1.0 (ref)		1.0 (ref)	
⩾22.5, <27.7	0.97 (0.76, 1.23)	0.7956	0.89 (0.70, 1.14)	0.3641	1.05 (0.81, 1.37)	0.6921
⩾27.7, <33.7	1.00 (0.79, 1.27)	0.9750	0.95 (0.75, 1.20)	0.6499	1.06 (0.81, 1.37)	0.6715
⩾33.7	1.61 (1.29, 2.00)	<0.0001	1.55 (1.24, 1.93)	<0.0001	1.37 (1.07, 1.77)	0.0139
*P* trend	<0.0001		<0.0001		0.0121	
*90-day all-cause mortality*						
Tertiles						
<24.4	1.0 (ref)		1.0 (ref)		1.0 (ref)	
⩾24.4, <31.4	1.03 (0.87, 1.24)	0.7060	0.96 (0.81, 1.15)	0.6937	1.13 (0.93, 1.37)	0.2152
⩾34.4	1.45 (1.23, 1.72)	<0.0001	1.45 (1.23, 1.72)	<0.0001	1.41 (1.16, 1.72)	0.0005
*P* trend	<0.0001		<0.0001		0.0004	
Quartiles						
<22.5	1.0 (ref)		1.0 (ref)		1.0 (ref)	
⩾22.5, <27.7	1.00 (0.81, 1.23)	0.9912	0.92 (0.74, 1.13)	0.4073	1.03 (0.82, 1.30)	0.7740
⩾27.7, <33.7	1.11 (0.90, 1.35)	0.3352	1.03 (0.84, 1.27)	0.7506	1.14 (0.91, 1.43)	0.2464
⩾33.7	1.65 (1.36, 1.99)	<0.0001	1.60 (1.32, 1.94)	<0.0001	1.43 (1.15, 1.79)	0.0016
*P* trend	<0.0001		<0.0001		0.0007	
*365-day all-cause mortality*						
Tertiles						
<24.4	1.0 (ref)		1.0 (ref)		1.0 (ref)	
⩾24.4, <31.4	1.11 (0.95, 1.29)	0.2009	1.04 (0.89, 1.21)	0.6544	1.18 (0.99, 1.40)	0.0595
⩾34.4	1.47 (1.26, 1.70)	<0.0001	1.50 (1.29, 1.74)	<0.0001	1.44 (1.21, 1.71)	<0.0001
*P* trend	<0.0001		<0.0001		<0.0001	
Quartiles						
<22.5	1.0 (ref)		1.0 (ref)		1.0 (ref)	
⩾22.5, <27.7	1.02 (0.85, 1.22)	0.8577	0.93 (0.77, 1.11)	0.4153	1.02 (0.83, 1.25)	0.8496
⩾27.7, <33.7	1.08 (0.91, 1.30)	0.3705	1.02 (0.85, 1.22)	0.8299	1.10 (0.90, 1.34)	0.3550
⩾33.7	1.61 (1.36, 1.91)	<0.0001	1.61 (1.35, 1.90)	<0.0001	1.41 (1.16, 1.73)	0.0007
*P* trend	<0.0001		<0.0001		0.0003	

HR, hazard ratio; CI, confidence interval.

Models were derived from Cox proportional hazards regression models.

Non-adjusted model adjust for: none.

Adjust I model adjust for: age, ethnicity and gender.

Adjust II model adjust for: age, gender, ethnicity, systolic blood pressure, diastolic blood pressure, temperature, SPO_2_, anion gap, bicarbonate, chloride, haemoglobin, lactate, platelet, APTT, PT, BUN, WBC, vasopressor use, atrial fibrillation, liver disease, respiratory failure, SOFA, SAPSII.

### Subgroup analyses

Subgroup analysis of the associations between NPAR and 90-day all-cause mortality was performed (Table 3), and there were no interactions in most strata (*P* = 0.0697–0.8841). Patients with a sodium ⩾136 mmol/l had a significantly higher risk of 90-day mortality with a NPAR ⩾31.4 (HR 1.89, 95% CI 1.49–2.40, *P* = 0.0354). Similarly, patients with a chloride ⩾102 mmol/l, WBC ⩾10.3 × 10^9^/l, haematocrit ⩾28.7% and haemoglobin ⩾9.5 g/dl showed an increased risk with a NPAR ⩾31.4 (HR, 95% CI: 1.72, 1.35–2.18; 1.74, 1.34–2.25; 1.81, 1.40–2.35; 1.76, 1.36–2.27, respectively).

**Table 3.** Subgroup analysis of the associations between the NPARs and 90-day all-cause mortality

	No. of patients	NPARs			*P* for interaction
		<24.4	⩾24.4, <31.4	⩾31.4	
CHF					0.1582
No	1750	1.0 (ref)	1.02 (0.83, 1.24)	1.37 (1.14, 1.65)	
Yes	416	1.0 (ref)	0.86 (0.57, 1.30)	1.84 (1.23, 2.76)	
AFIB					0.8613
No	1496	1.0 (ref)	0.94 (0.76, 1.18)	1.38 (1.12, 1.70)	
Yes	670	1.0 (ref)	1.02 (0.75, 1.38)	1.56 (1.16, 2.09)	
CAD					0.1415
No	1764	1.0 (ref)	0.95 (0.78, 1.16)	1.33 (1.11, 1.60)	
Yes	402	1.0 (ref)	1.08 (0.70, 1.65)	2.07 (1.38, 3.10)	
Stroke					0.0882
No	2047	1.0 (ref)	0.96 (0.80, 1.16)	1.50 (1.26, 1.79)	
Yes	119	1.0 (ref)	1.02 (0.55, 1.88)	0.76 (0.39, 1.51)	
Malignancy					0.6360
No	1698	1.0 (ref)	0.98 (0.80, 1.21)	1.36 (1.10, 1.66)	
Yes	468	1.0 (ref)	1.01 (0.72, 1.43)	1.60 (1.19, 2.16)	
Liver disease					0.0203
No	1890	1.0 (ref)	1.01 (0.83, 1.24)	1.62 (1.34, 1.96)	
Yes	276	1.0 (ref)	0.79 (0.54, 1.17)	0.89 (0.61, 1.30)	
Renal disease					0.7984
No	1788	1.0 (ref)	0.95 (0.78, 1.17)	1.46 (1.21, 1.77)	
Yes	378	1.0(ref)	0.98 (0.68, 1.43)	1.36 (0.94, 1.98)	
Respiratory failure					<0.0001
No	889	1.0 (ref)	1.22 (0.85, 1.74)	2.66 (1.91, 3.72)	
Yes	1277	1.0 (ref)	0.88 (0.72, 1.08)	1.10 (0.90, 1.34)	
Pneumonia					0.0235
No	1270	1.0 (ref)	0.77 (0.60, 0.99)	1.29 (1.03, 1.60)	
Yes	896	1.0 (ref)	1.27 (0.98, 1.64)	1.72 (1.33, 2.23)	
COPD					0.2817
No	2106	1.0 (ref)	0.95 (0.79, 1.13)	1.44 (1.21, 1.70)	
Yes	60	1.0 (ref)	1.88 (0.63, 5.60)	4.09 (1.06, 15.71)	
ARDS					0.3307
No	2106	1.0 (ref)	0.96 (0.80, 1.15)	1.44 (1.22, 1.71)	
Yes	60	1.0 (ref)	1.93 (0.55, 6.78)	3.61 (1.10, 11.90)	
Vasopressor use					0.0019
No	775	1.0 (ref)	1.22 (0.85, 1.74)	2.41 (1.72, 3.36)	
Yes	1391	1.0 (ref)	0.87 (0.71, 1.07)	1.15 (0.95, 1.40)	
Albumin, g/dl					0.1726
<2.8	963	1.0 (ref)	0.64 (0.45, 0.89)	0.71 (0.54, 0.93)	
⩾2.8	1203	1.0 (ref)	1.03 (0.83, 1.27)	1.44 (0.96, 2.16)	
Neutrophil percentage, %					0.0775
<82	1064	1.0 (ref)	0.94 (0.74, 1.18)	1.39 (1.08, 1.78)	
⩾82	1102	1.0 (ref)	1.44 (1.00, 2.07)	2.29 (1.61, 3.24)	
Sodium, mmol/l					0.0354
<136	915	1.0 (ref)	0.83 (0.64, 1.08)	1.09 (0.85, 1.38)	
⩾136	1251	1.0 (ref)	1.15 (0.90, 1.48)	1.89 (1.49, 2.40)	
Potassium, mmol/l					0.1088
<3.7	998	1.0 (ref)	0.73 (0.55, 0.98)	1.29 (1.00, 1.66)	
⩾3.7	1168	1.0 (ref)	1.15 (0.92, 1.44)	1.60 (1.28, 2.00)	
Chloride, mmol/l					0.0039
<102	1006	1.0 (ref)	0.97 (0.76, 1.24)	1.25 (0.98, 1.59)	
⩾102	1160	1.0 (ref)	0.97 (0.74, 1.26)	1.72 (1.35, 2.18)	
WBC, 10^9^/l					0.0044
<10.3	1077	1.0 (ref)	0.79 (0.62, 1.01)	1.24 (0.97, 1.59)	
⩾10.3	1089	1.0 (ref)	1.24 (0.94, 1.63)	1.74 (1.34, 2.25)	
Platelet, 10^9^/l					0.0173
<162	1080	1.0 (ref)	0.98 (0.77, 1.23)	1.27 (1.02, 1.59)	
⩾162	1086	1.0 (ref)	1.08 (0.81, 1.43)	1.86 (1.42, 2.43)	
Haematocrit, %					0.0255
<28.7	1080	1.0 (ref)	0.80 (0.62, 1.04)	1.16 (0.93, 1.45)	
⩾28.7	1086	1.0 (ref)	1.15 (0.89, 1.48)	1.81 (1.40, 2.35)	
Haemoglobin, g/dl					0.0430
<9.5	1046	1.0 (ref)	0.89 (0.69, 1.16)	1.18 (0.94, 1.48)	
⩾9.5	1119	1.0 (ref)	1.04 (0.81, 1.34)	1.76 (1.36, 2.27)	
Creatinine, mEq/l					0.0432
<1.3	1080	1.0 (ref)	0.97 (0.72, 1.29)	1.38 (1.06, 1.81)	
⩾1.3	1086	1.0 (ref)	0.95 (0.75, 1.19)	1.50 (1.21, 1.86)	
BUN, mg/dl					0.8841
<28	1050	1.0 (ref)	1.01 (0.75, 1.38)	1.47 (1.10, 1.96)	
⩾28	1116	1.0 (ref)	0.89 (0.72, 1.11)	1.36 (1.11, 1.68)	
Anion gap, mmol/l					0.2113
<13	856	1.0 (ref)	0.82 (0.58, 1.15)	1.37 (1.01, 1.86)	
⩾13	1310	1.0(ref)	1.04 (0.84, 1.28)	1.57 (1.28, 1.92)	
Bicarbonate, mg/dl					0.1203
<19	981	1.0 (ref)	0.81 (0.63, 1.03)	1.13 (0.90, 1.42)	
⩾19	1185	1.0 (ref)	1.18 (0.91, 1.54)	1.84 (1.43, 2.38)	
Lactate, mmol/l					0.7467
<1.6	887	1.0 (ref)	0.99 (0.71, 1.39)	1.49 (1.08, 2.05)	
⩾1.6	1042	1.0 (ref)	1.15 (0.92, 1.44)	1.62 (1.31, 2.00)	
Glucose, mg/dl					0.7137
<134	1078	1.0 (ref)	0.92 (0.72, 1.17)	1.35 (1.07, 1.71)	
⩾134	1079	1.0 (ref)	1.01 (0.78, 1.31)	1.55 (1.21, 1.98)	
Bilirubin, mg/dl					0.0045
<0.7	936	1.0 (ref)	0.79 (0.58, 1.07)	1.69 (1.28, 2.23)	
⩾0.7	1060	1.0 (ref)	1.09 (0.87, 1.36)	1.21 (0.97, 1.52)	
PT, second					0.0697
<14.7	1029	1.0 (ref)	0.84 (0.62, 1.15)	1.72 (1.31, 2.27)	
⩾14.7	1080	1.0 (ref)	1.03 (0.82, 1.28)	1.23 (0.99, 1.52)	
APTT, second					0.4212
<31.2	1046	1.0 (ref)	1.00 (0.76, 1.33)	1.64 (1.25, 2.15)	
⩾31.2	1061	1.0 (ref)	0.96 (0.76, 1.21)	1.20 (0.97, 1.49)	
INR					0.2165
<1.3	781	1.0 (ref)	0.85 (0.61, 1.20)	1.71 (1.25, 2.36)	
⩾1.3	1328	1.0 (ref)	1.01 (0.82, 1.24)	1.29 (1.06, 1.58)	
SBP, mmHg					0.4607
<108	1075	1.0 (ref)	0.85 (0.67, 1.07)	1.25 (1.00, 1.55)	
⩾108	1082	1.0 (ref)	1.15 (0.88, 1.51)	1.63 (1.24, 2.13)	
DBP, mmHg					0.2048
<57	1077	1.0 (ref)	0.94 (0.74, 1.20)	1.54 (1.22, 1.93)	
⩾57	1080	1.0 (ref)	0.97 (0.75, 1.27)	1.26 (0.97, 1.63)	
MBP, mmHg					0.5355
<72	1078	1.0 (ref)	1.07 (0.84, 1.37)	1.71 (1.36, 2.14)	
⩾72	1080	1.0 (ref)	0.83 (0.64, 1.08)	1.04 (0.80, 1.35)	
Heart rate, beats/minute					0.0803
<91	1079	1.0 (ref)	1.15 (0.88, 1.49)	1.91 (1.49, 2.46)	
⩾91	1079	1.0 (ref)	0.83 (0.65, 1.06)	1.09 (0.87, 1.38)	
Respiratory rate, beats/minute					0.0136
<21	1078	1.0 (ref)	1.14 (0.86, 1.51)	1.85 (1.42, 2.41)	
⩾21	1078	1.0 (ref)	0.82 (0.65, 1.03)	1.18 (0.95, 1.48)	
Temperature, °C					0.7159
<36.8	1073	1.0 (ref)	0.88 (0.69, 1.12)	1.52 (1.22, 1.90)	
⩾36.8	1074	1.0 (ref)	1.02 (0.78, 1.33)	1.25 (0.96, 1.62)	
SPO2, %					0.7942
<97.2	1076	1.0 (ref)	0.87 (0.69, 1.10)	1.35 (1.08, 1.69)	
⩾97.2	1077	1.0 (ref)	1.13 (0.85, 1.50)	1.71 (1.32, 2.23)	
RRT					0.0292
No	1845	1.0 (ref)	0.99 (0.81, 1.21)	1.59 (1.32, 1.92)	
Yes	321	1.0 (ref)	0.80 (0.54, 1.19)	0.90 (0.62, 1.32)	

CHF, congestive heart failure; AFIB, atrial fibrillation; CAD, coronary artery disease; COPD, chronic obstructive pulmonary disease; ARDS, acute respiratory distress syndrome; WBC, white blood cell; BUN, blood urea nitrogen; PT, prothrombin time; APTT, activated partial thromboplastin time; INR, international normalised ratio; SBP, systolic blood pressure; DBP, diastolic blood pressure; MBP, mean blood pressure; RRT, renal replacement therapy.

The modification and interaction of subgroup were inspected by the likelihood ration test.

## Discussion

Our main findings can be summarised as follows. First, higher NPAR was associated with increased risk of 30-, 90- and 365-day all-cause mortality in critically ill patients with severe sepsis or septic shock after adjustments for age, ethnicity and gender. Furthermore, after adjustments for more potential confounders, higher NPAR remained significantly associated with all-cause mortality. To our knowledge, this was the first study to investigate the prognostic value of NPAR in critically ill patients with severe sepsis or septic shock; we found that higher NPAR was a novel predictor of poorer prognosis, and it was a better predictor than either albumin or neutrophil percentage alone.

Clinically, we found a phenomenon in which the high neutrophil percentage and the low albumin levels are associated with poor outcomes in patients with severe sepsis or septic shock. Previous studies focused on the neutrophil-to-albumin ratio, mainly in significantly predicting prognosis of palliative pancreatic cancer treatment and rectal cancer [16, 17]. Neutrophil percentage can be used as a practical marker to assess inflammation, and the serum neutrophil percentage and inflammatory cytokines are increased in infected patients [24, 25]. Moreover, previous studies have described the relationship between hypoproteinemia and mortality in stroke, myocardial infarction and hip fracture [26–28]. These findings suggested that lower albumin values correlated with poorer prognosis of the disease. On the other hand, lower albumin values correlated with higher the values of NPAR. In our study, by comparing changes in NPAR values of patients with severe sepsis or septic shock, we found that increasing values of NPAR predicted poor sepsis prognosis. Russell *et al*., [29] showed that peripheral blood leucocyte ratios are useful biomarkers for infection. In critical illness due to sepsis, there is a signal and prognosis associated with NLR, and longitudinal measurements of these biomarkers during infection could be informative. Our findings also indicated that NPAR and NLR had similar predictive abilities for poor outcomes.

Neutrophils are part of the differential of WBC counts that are typically sensitive to bacterial and fungal infections [30]. Walling *et al*., [31] demonstrated that neutrophil percentages above 80% provided a good distinction between positive and negative blood cultures among sepsis patients. However, the role of neutrophils in predicting bloodstream infection remained questionable, because stress, medication, trauma and abnormal bone marrow formation could cause these changes [32, 33]. Therefore, looking for a simple and reliable clinical predictor of mortality in sepsis is significant. Albumin levels reflect nutritional status and organ function, and the underlying inflammatory state give rise to a decrease of albumin production in liver by increasing inflammatory factors, the primary cause of hypoalbuminemia that occurs early in sepsis [34, 35]. Therefore, based on our findings, NPAR, a new biomarker composed of neutrophil percentage and albumin that closely related to the inflammatory response, can significantly predict the prognosis of sepsis.

Our study had some limitations. First, the study was a single-centre retrospective design, and was therefore subject to selection bias. Second, we extracted NPAR in patients only upon admission to the ICU and did not assess changes during the ICU stay. Third, this database does not use the latest sepsis definitions (sepsis 3.0), and severe sepsis no longer forms part of the sepsis 3.0 definitions, this may affect the conclusion. Fourth, missing the aetiology of sepsis and specific cause of death in the MIMIC database failed to make the study more detailed and comprehensive. Fifth, although we have done our best to use a multivariate model to control bias, there remain numerous other known and unknown factors. Furthermore, the database contains a few inaccurate data elements. Therefore, multi-centre prospective studies are needed to confirm these findings.

## Conclusions

Our findings demonstrated that higher NPAR was associated with increased risk of all-cause mortality in critically ill patients with severe sepsis or septic shock. Nevertheless, the conclusions need to be confirmed in large prospective multicentre studies.

## Supplementary material

For supplementary material accompanying this paper visit https://doi.org/10.1017/S0950268820000771. click here to view supplementary material

## Data

The clinical data used to support the findings of this study were supplied by Monitoring in Intensive Care Database III version 1.4 (MIMIC-III v.1.4). Although the database is publicly and freely available, researchers must complete the National Institutes of Health's web-based course known as Protecting Human Research Participants to apply for permission to access the database.
