# Supplementary material for: Increased neutrophil percentage-to-albumin ratio is associated with all-cause mortality in patients with severe sepsis or septic shock
Source: Epidemiol Infect. 2020 Apr 2;148:e87. doi: 10.1017/S0950268820000771 (PMC7189348; doi:10.1017/S0950268820000771)
Supplement: Supplementary file 1 [file S0950268820000771sup001.docx]

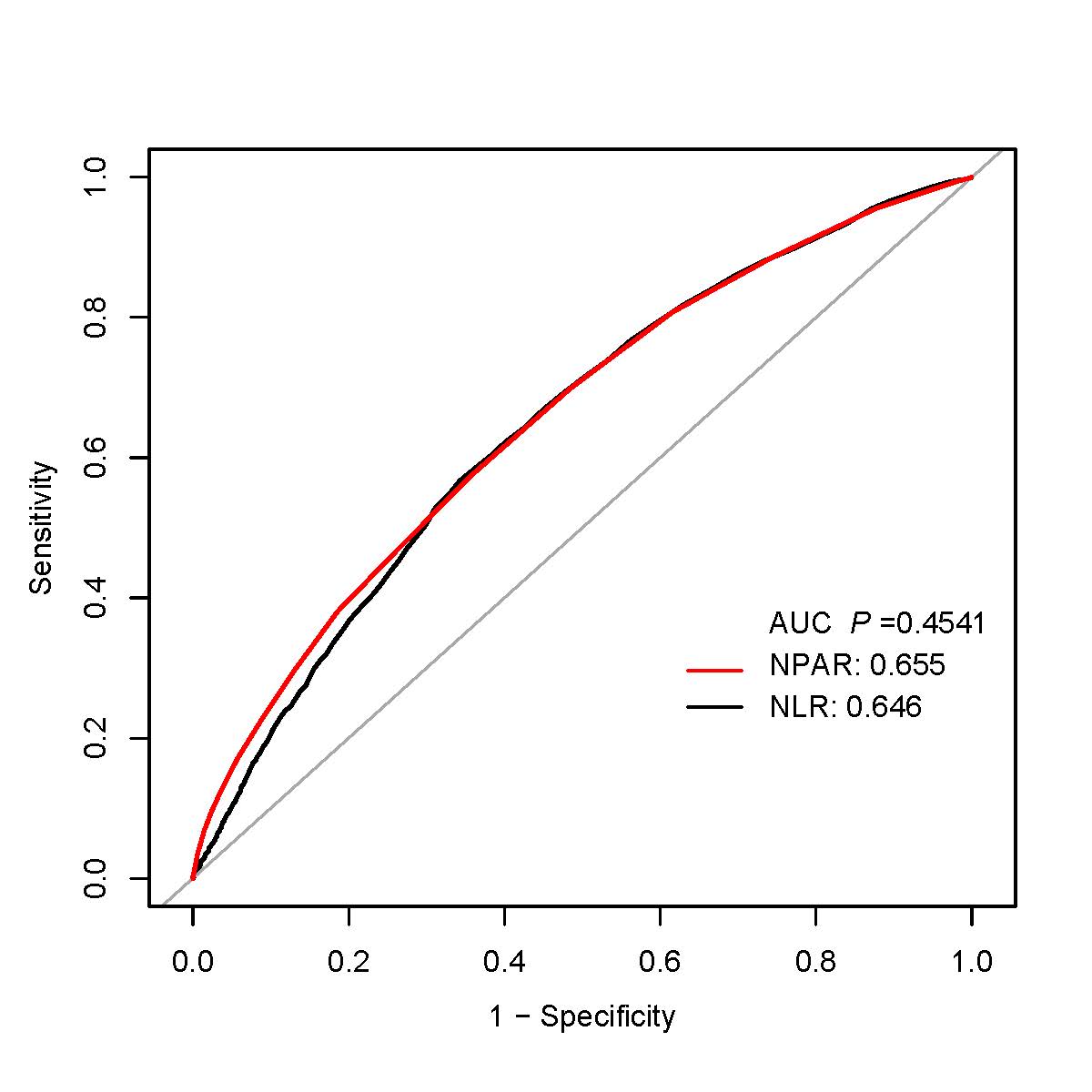


Fig. 1 ROC curves for the prediction of mortality in critically ill patients with severe sepsis or septic shock, the ability of NPAR and NLR to predict 365-day mortality. The AUCs for NPAR and NLR were 0.655 and 0.646, respectively.


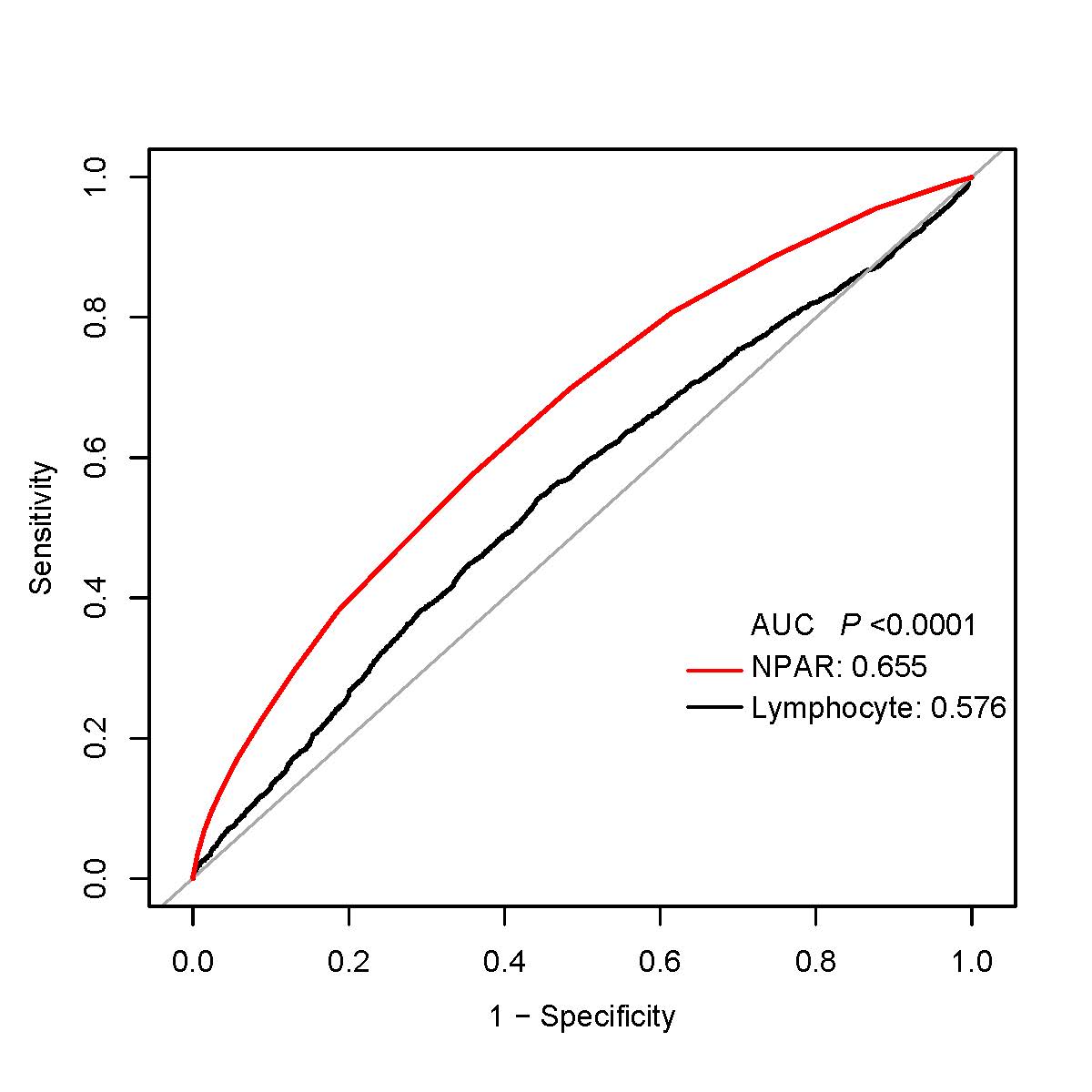


Fig. 2 ROC curves for the prediction of mortality in critically ill patients with severe sepsis or septic shock, the ability of NPAR and lymphocyte to predict 365-day mortality. The AUCs for NPAR and lymphocyte were 0.655 and 0.576, respectively.
